# DNAJB9 suppresses the metastasis of triple-negative breast cancer by promoting FBXO45-mediated degradation of ZEB1

**DOI:** 10.1038/s41419-021-03757-x

**Published:** 2021-05-08

**Authors:** Hye-Youn Kim, Young-Mi Kim, Suntaek Hong

**Affiliations:** 1 Department of Biochemistry, Lee Gil Ya Cancer and Diabetes Institute, Gachon University College of Medicine, Incheon, 21999 Republic of Korea; 2 Department of Health Sciences and Technology, GAIHST, Gachon University, Incheon, 21999 Republic of Korea

**Keywords:** Breast cancer, Metastasis, Ubiquitylation, Tumour-suppressor proteins

## Abstract

DNAJB9, a member of the heat shock protein 40 family, acts as a multifunctional player involved in the maintenance of their client proteins and cellular homeostasis. However, the mechanistic action of DNAJB9 in human malignancies is yet to be fully understood. In this study, we found that ectopic restoration of DNAJB9 inhibits the migration, invasion, in vivo metastasis, and lung colonization of triple-negative breast cancer (TNBC) cells. Mechanistically, DNAJB9 stabilizes FBXO45 protein by suppressing self-ubiquitination and reduces the abundance of ZEB1 by Lys48-linked polyubiquitination to inhibit the epithelial–mesenchymal transition (EMT) and metastasis. Clinically, the reduction of DNAJB9 expression, concomitant with decreased FBXO45 abundance in breast cancer tissues, correlates with poorer clinical outcomes of patients with breast cancer. Taken together, our results provide a novel insight into the metastasis of TNBC and define a promising therapeutic strategy for cancers with overactive ZEB1 by regulating the DNAJB9–FBXO45 signaling axis.

## Introduction

Breast cancer represents the most common cancer among women and the fifth leading cause of cancer death worldwide^1^. Breast cancer is classified into four subtypes: HER2-enriched, luminal A, luminal B, and triple-negative breast cancer (TNBC)^2,3^. Accounting for 10–20% of all breast cancers, TNBC is characterized by the lack of estrogen receptor, progesterone receptor, and epidermal growth factor receptor 2 expression levels and has the worst outcome due to unsatisfactory therapeutic efficacy and high risk of distant metastases and mortality^4,5^. Notably, compared with other subtypes, TNBC cells have an activated epithelial–mesenchymal transition (EMT) program, a highly dynamic process by which well-polarized epithelial cells are converted into non-polarized mesenchymal cells, including alterations in cell–cell and cell–matrix adhesion, remodeling of the cytoskeleton, and loss of polarities, along with enhanced cell mobility and invasiveness, higher metastatic potential, and resistance to effective therapies^6–9^. Standard chemotherapy and radiation regimens have been the only accepted treatment options for women with TNBC to increase the overall survival (OS) rate. However, these systemic treatments have adverse side effects and usually fail, resulting in higher rates of recurrence and short OS^10,11^. Therefore, the clinical requirement of TNBC remains a major challenge, and understanding of the molecular mechanisms in the biology and pathogenesis of TNBC may help in identifying novel strategies for the prevention of cancer and the development of more effective treatment.

DnaJ is a member of the heat shock protein 40 (hsp40) family of molecular chaperones and is important for protein folding, unfolding, translation, degradation, and intracellular signaling functions^12^. Recent studies have demonstrated that some hsp40 proteins function as tumor suppressors, including DNAJB6^13,14^, DNAJB4^15–18^, and DNAJA3^19,20^. In addition, DNAJB9 belongs to the type II DnaJ homolog subfamily B, containing an N-terminal J domain and a G/F-rich region in the middle and a C-terminal domain, which was shown to interact with its substrate protein^21,22^. DNAJB9 inhibits the proapoptotic function of p53 as a negative feedback regulator^23^. Moreover, a recent study has shown that DNAJB9, a luminal co-chaperone, is a novel therapeutic target for cystic fibrosis due to its involvement in cystic fibrosis transmembrane conductance regulator–endoplasmic reticulum-associated protein degradation^24^. However, the cellular function of DNAJB9 in development and metastasis of breast cancer remains largely unknown.

In this study, we showed that DNAJB9 acts as a negative regulator of ZEB1 by stabilizing FBXO45 ubiquitin ligase by inhibiting self-ubiquitination and suppresses the metastasis of TNBC in in vitro and in vivo models. Moreover, DNAJB9 is downregulated in TNBC tissues, and low expression of DNAJB9 predicts the aggressive phenotype of breast cancer cells and poor prognosis. These findings could offer a novel therapeutic strategy for treating metastatic breast cancer by regulating the DNAJB9–FBXO45 signaling axis.

## Materials and methods

### Cell culture and reagents

The human TNBC cell lines, Hs578T, MDA-MB-231, and MDA-MB-157 (ATCC), were cultured using Dulbecco’s Modified Eagle’s Medium supplemented with penicillin/streptomycin and 10% fetal bovine serum (FBS). The human luminal cell lines, MCF7, BT474, and T47D (ATCC), were cultured in RPMI-1640 supplemented with penicillin/streptomycin and 10% FBS. All cells were maintained at 37 °C in a 5% CO_2_ incubator. The cell lines in this study were routinely tested for mycoplasma contamination by polymerase chain reaction (PCR). Antibodies against the following proteins were used as indicated: E-cadherin (24E10; Cell Signaling Technology, Danvers, MA), Vimentin (5741; Cell Signaling Technology), Snail (3879; Cell Signaling Technology), MYC (9E10; Santa Cruz Biotechnology, Dallas, TX), ZEB1 (H-102; Santa Cruz Biotechnology), β-actin (A5441; Sigma-Aldrich, St. Louis, MO), DNAJB9 (13157-1-AP; Proteintech, IL, USA), and FBXO45 (PA5-49458; Invitrogen, Carlsbad, CA).

### Animal study

The in vivo metastasis models were established with 6–8-week-old immunodeficient BALB/c athymic female nude mice (Orient Bio, Seongnam, Korea). All experiments were conducted following the guidelines and protocols approved by the Institutional Animal Care and Use Committee of the Lee Gil Ya Cancer and Diabetes Institute, Gachon University (Incheon, Korea). The mice were randomly separated into five groups. MDA-MB-231 cells with DNAJB9-OE, DNAJB9-OE–FBXO45-KD, and empty vector control were harvested, and 1 × 10^6^ cells were intravenously injected into the nude mice (5 animals per group). Metastasis incidence was measured and monitored using the IVIS Spectrum in vivo imaging system (Caliper Life Sciences, Hopkinton, MA) weekly by intraperitoneally injecting 150 mg/ml luciferin reagent (Promega, Madison, WI)^25^. The intensity of the metastasized tumors was measured using Living Image (V.3.1.0; Caliper Life Sciences). After 10 weeks, the mice were euthanized, their lung tissues were removed and fixed in 10% formalin, and lung metastases were evaluated under a microscope after hematoxylin and eosin staining. Tumor areas were measured using ImageJ (National Institutes of Health, Bethesda, MD). When mice showed severe weight loss or behavior abnormalities, they were euthanized.

### Immunoblotting and immunoprecipitation

Cells were lysed using an NP-40 lysis buffer (50 mmol/l Tris (pH 7.5), 150 mmol/l NaCl, 10% glycerol, 0.5% Nonidet P-40, and protease inhibitors) for 30 min at 4 °C, and cell debris was removed by centrifugation at 10,000 × *g* for 10 min at 4 °C. The protein concentrations were determined using the bicinchoninic acid method (Thermo Scientific, Rockford, IL) with bovine serum albumin (BSA) as a standard. For detecting target protein, an equal number of protein lysates were separated using sodium dodecyl sulfate (SDS)–polyacrylamide gel electrophoresis, transferred to an Immobilon PVDF membrane (Millipore, Bedford, MA), and then probed with specific primary antibodies. Specific proteins were visualized using chemiluminescent reagents according to the manufacturer’s instructions (Thermo Scientific). For immunoprecipitation, the protein extracts were incubated with specific antibodies overnight at 4 °C and treated with protein A/G-agarose beads for 1 h at 4 °C. The precipitated protein complexes were removed by boiling for 5 min in 2× Tris-glycine SDS sample buffer and were detected using immunoblotting.

### The generation of overexpressed (OE) or knockdown (KD) cells

For the generation of OE cells, a pCAG–lentiviral vector (empty control, Flag-tagged DNAJB9, or FBXO45) was co-transfected into Lenti293 cells with packaging DNAs using polyethylenimine. For KD cells, a pLKO–lentiviral vector (shGL2 control, shDNAJB9, shFBXO45, or shSIAH1) was co-transfected into Lenti293 cells. Supernatants containing viral particles were collected 48 h post-transfection, centrifuged to remove cell debris, and filtered through 0.45-µm filters. The lentiviruses were used to infect target cells cultured in a medium containing 10 µg/ml polybrene (Sigma-Aldrich). Infected cells were then selected in the presence of 1 µg/ml puromycin. The target cells were infected with lentiviral supernatants 3 times every 12 h, and the protein expression was evaluated using western blotting and quantitative reverse transcription–PCR (qRT–PCR). The short hairpin RNA (shRNA) sequences used in this study are listed in Supplementary Table S1.

### RNA extraction, reverse transcription PCR, and real-time RT-PCR

Total RNA was prepared using TRIzol solution (Invitrogen), as described by the manufacturer’s instructions. RNA was converted to cDNA by reverse transcription using a PrimeScript II RT Reagent Kit (Takara, Kyoto, Japan). SYBR-Green Premix Ex-Tag™ (Takara) was used for quantifying gene expression using real-time quantitative PCR using the Prism 7900HT sequence detection system (Thermo Scientific) according to manufacturers’ protocols. Oligonucleotides used for the analysis of gene expression are listed in Supplementary Table S2. The expression of each gene was quantitated using the 2^−∆∆CT^ method and normalized using cyclophilin. Data were analyzed in at least triplicates and expressed as mean ± standard deviation (SD).

### Wound healing and invasion assays

To determine the migration ability of breast cancer cells, 0.3 × 10^4^ cells were seeded and maintained until confluence in 6-well plates. After scratching with a pipette tip, the cells were cultured in a complete medium. Migrated cells were quantified at 24 h after wounding. To determine the invasion ability, 5 × 10^4^ cells were seeded in the upper chamber (8-μm inserts; Thermo Scientific) in a 200 μl serum-free medium. After 24 h, the cells that invaded to the bottom of the membrane were fixed with 4% formaldehyde and stained using 0.005% crystal violet (Sigma-Aldrich). The area of migrated and invaded cells in each well were quantified by counting at least four randomly chosen fields using a bright-field microscope.

### Immunohistochemistry (IHC) and immunocytochemistry

Isolated tumor tissues were fixed in 4% neutral-buffered formalin, embedded, and then sectioned according to standard protocols. After treatment with 0.03% hydrogen peroxide, slides were microwaved for 10 min in a 10 mM citrate buffer (pH 6.0) with 0.01% Tween 20, allowed to cool for 10 min, and stained with specific antibodies. Then antibodies were visualized using a diaminobenzidine reagent, whereas the nuclei were counterstained with hematoxylin QS (H-3404; Vector Laboratories, Burlingame, CA). For immunocytochemistry, each cell line was seeded in four-well chamber slides. After 24 h of incubation, the cells were fixed using 4% neutral-buffered formalin for 15 min at room temperature, washed 3 times, and permeabilized with 1% Triton X-100/phosphate-buffered saline (PBS). After the cells were washed 3 times, they were blocked with blocking buffer (1% BSA/PBS) for 1 h. Then primary antibody (1:100) was added to each well and incubated overnight at 4 °C. After washing out the primary antibody solution, the cells were incubated with Alexa Fluor 594-conjugated (A32740, Invitrogen) secondary antibody for 1 h at room temperature and nuclei counterstained with 4,6-diamidino-2-phenylindole for 1 min.

### Ubiquitination assay

For in vitro ubiquitination experiments, relevant plasmids were transfected into HEK293 cells and then treated with 10 µM proteasome inhibitor MG132 (Calbiochem, Bedford, MA) for 6 h. These cells were lysed using a NP-40 lysis buffer and incubated with the indicated primary antibodies at 4 °C for 16 h. For the immunoprecipitation of ubiquitinated proteins, lysates were incubated with protein A/G-agarose beads at 4 °C for 1 h. Then the beads were washed with PBS three times and analyzed using western blotting. For the immunoprecipitation of ubiquitinated proteins, lysates were incubated with anti-FBXO45 antibody and protein A/G-agarose beads at 4 °C for 16 h. Then the immunoprecipitates were separated using SDS–polyacrylamide gel electrophoresis and analyzed with ubiquitin antibody.

### Protein half-life analysis

For the analysis of ZEB1 half-life, MDA-MB-231 cells with or without DNAJB9 OE were incubated with 10 µM cycloheximide (CHX) for the indicated time points, and endogenous ZEB1 was detected using western blotting^26^. The relative concentration of proteins at time 0 was defined as 100%. The level of proteins was quantified by the normalization of β-actin protein.

### Online public dataset analysis

For meta-analysis, online tools including the Gene Expression Omnibus (GEO) DataSets (National Center for Biotechnology Information, Bethesda, MD), Oncomine (https://www.oncomine.org), and the Gene Expression database of Normal and Tumor Tissue (GENT) (http://medical-genome.kribb.re.kr/GENT/) were used to compare the expression of DNAJB9 between various cancers and their normal counterparts. The gene expression and clinical profiles of patients with breast cancer were downloaded from LinkedOmics (http://www.linkedomics.org), which is an online public dataset containing multi-omics data of breast cancer from The Cancer Genome Atlas project^27^.

### Statistical analyses

The statistical significance of the differences between groups was determined using Student’s *t* test (two tailed), and error bars show the SD of the mean. Survival probabilities were determined using the Kaplan–Meier method and were analyzed using log-rank tests. Statistical analyses of all data were conducted using Prism (version 5.0; GraphPad Software, San Diego, CA)^28^. Multivariate Cox regression analysis was performed to assess the prognostic value of DNAJB9 and independent prognostic factors. Data are presented as mean ± SD unless otherwise stated. In all statistical tests, *p* values of <0.05 were used to denote statistical significance. There were no studies in which investigators were blinded, and all experiments were repeated at least three times. The sample size was chosen on the basis of literature in this field.

## Results

### The low expression of DNAJB9 in TNBC is correlated with poor clinical outcomes

**Fig. 1:**
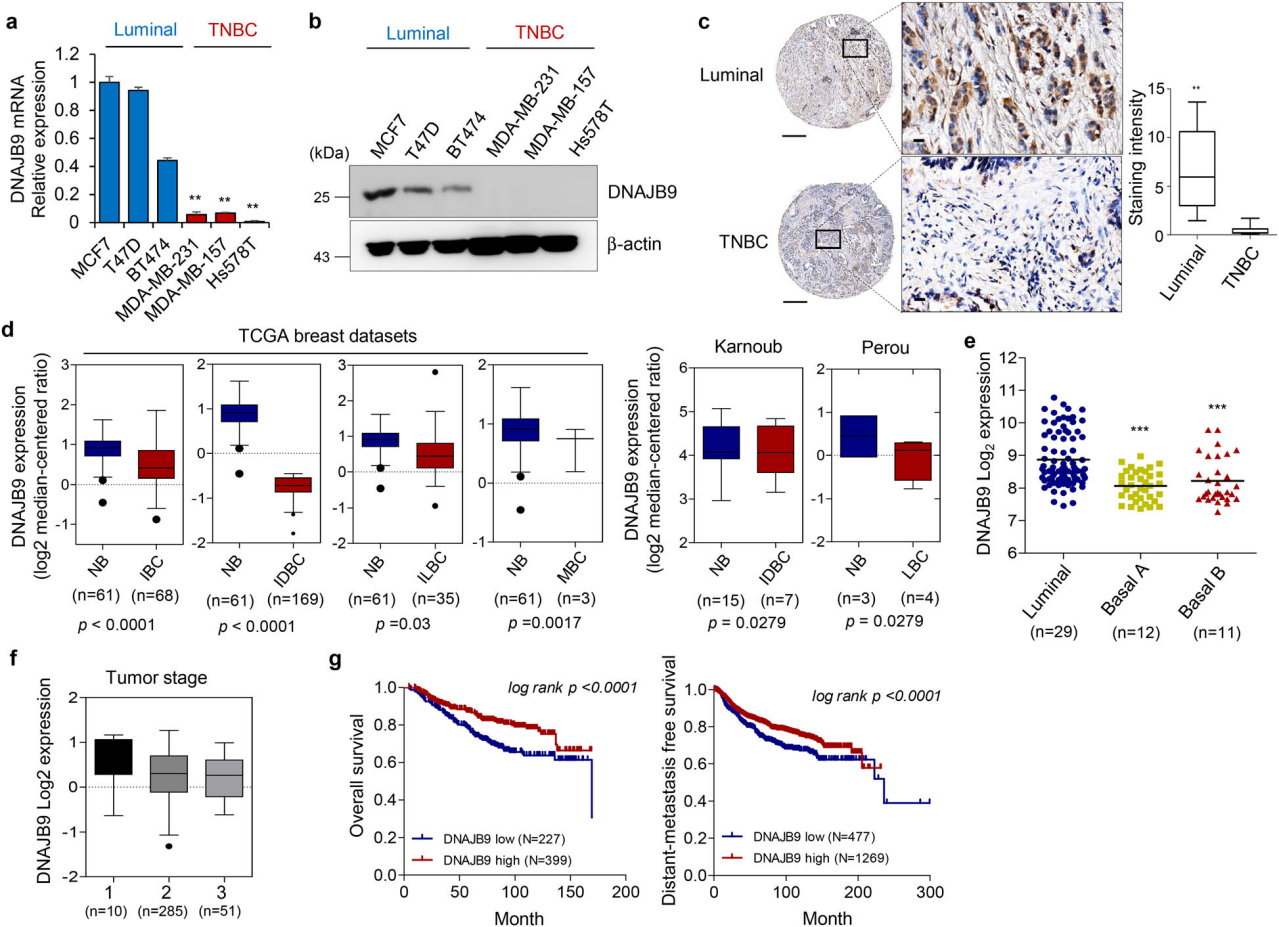
Low expression of DNAJB9 in TNBC is correlated with poor clinical outcomes. **a**, **b** To confirm the expression of DNAJB9 at mRNA and protein levels, total RNAs and proteins were extracted from luminal and TNBC cell lines. Then the expression levels of DNAJB9 were determined using qRT–PCR or western blotting. Cyclophilin and β-actin were detected as normalization controls, respectively. **c** Representative immunohistochemical staining images of DNAJB9 in 26 cases of paraffin-embedded human breast cancer specimens. Images were quantified using ImageJ from at least three fields. Scale bar, 100 μm. Staining intensity was compared between luminal and TNBC cells. **d** Box plots of DNAJB9 mRNA in different subtypes of breast cancer and normal tissues obtained from the Oncomine public dataset. NB normal breast, IBC invasive breast cancer, IDBC invasive ductal breast cancer, ILBC invasive lobular breast cancer, MBC mucinous breast cancer, LBC lobular breast cancer. **e** Scatter dot plots of DNAJB9 mRNA transcript levels in a panel of 52 breast cancer cell lines using public microarray datasets. **f** DNAJB9 mRNA expression in breast tumors stratified based on the tumor stage using the Oncomine public dataset. **g** Kaplan–Meier analysis of the OS and DMFS of patients with breast cancer. Patients with tumors expressing DNAJB9 at levels higher than the mean value are labeled in red, whereas those with tumors with gene expression levels below the mean are shown in blue. *P* values were calculated using log-rank Mantel–Cox test. All *p* values were calculated using unpaired two-tailed Student’s *t* tests (**a**, **c**). Results are presented as mean ± standard deviation from three independent experiments. **p < 0.01; ***p < 0.001.

To investigate the function of DNAJB9 in breast cancer, the mRNA and protein expression levels of DNAJB9 were measured in luminal cell lines (MCF7, T47D, and BT474) and TNBC cell lines (MDA-MB-231, MDA-MB-157, and Hs578T) using qRT–PCR and western blotting. As a result, the mRNA and protein levels of DNAJB9 were significantly higher in luminal cell lines than those in TNBC cell lines (Fig. 1a, b). Next, we examined the expression level of DNAJB9 in 26 cases of paraffin-embedded human breast tumor specimens using IHC staining. Consistently, the expression of DNAJB9 was remarkably elevated in luminal tissues compared with TNBC tissues (Fig. 1c). Then we assessed the expression levels of DNAJB9 using the GENT database across diverse cancer and normal tissues. We observed that the dysregulation of DNAJB9 related to the corresponding normal tissue controls among different tumor types, including breast tumors (Supplementary Fig. S1a), indicate that DNAJB9 levels correlate inversely with the metastatic properties of breast cancer. To evaluate the relationship between DNAJB9 expression and clinical correlation, we further analyzed publicly available microarray datasets of patients with breast cancer in Oncomine. In line with our preclinical studies, DNAJB9 was consistently lower in breast tumor samples than in normal breast tissue (Fig. 1d). Analysis of 52 breast cancer cell lines using the GEO dataset (GSE41313) revealed that DNAJB9 transcripts were reduced in highly aggressive TNBC cell lines (basal A and B) compared to the less-aggressive luminal cell lines (Fig. 1e). Interestingly, Cox multivariate regression analysis demonstrated that low expression of DNAJB9 was an independent risk factor for shortened OS (*p* = 0.0032; hazard ratio (HR) = 0.71; 95% confidence interval (CI), 0.57–0.89) and distant metastasis-free survival (DMFS) (*p* = 0.0027; HR = 0.73; 95% CI, 0.6–0.9) (Supplementary Fig. S1b).

Next, we examined whether DNAJB9 expression could predict the tumor stage (T stage) of breast cancer. As expected, DNAJB9 was negatively correlated with the tumor stage of breast carcinoma tissue (Fig. 1f). Using the Kaplan–Meier plotter, we observed that patients with breast cancer with high DNAJB9 expression had improved OS and DMFS (*p* < 0.0001; Fig. 1g).

Studies have demonstrated that DNAJB4 (HLJ1) and DNAJB6 (MRJ) function as tumor suppressors in multiple types of cancer^15,29,30^. However, the expression levels of DNAJB4 and DNAJB6 in 52 breast cancer cell lines were significantly elevated in aggressive TNBC cell lines compared with DNAJB9 (Supplementary Fig. S2a). In addition, the expression levels of DNAJB4 and DNAJB6 showed no significant influence on the OS and DMFS of patients with breast cancer (Supplementary Fig. S2b). Taken together, our results demonstrated that DNAJB9 is lost in high-grade and aggressive breast cancers, is positively correlated with patient survival, and may act to suppress breast cancer progression and metastasis.

### DNAJB9 is a metastasis suppressor in TNBC

**Fig. 2:**
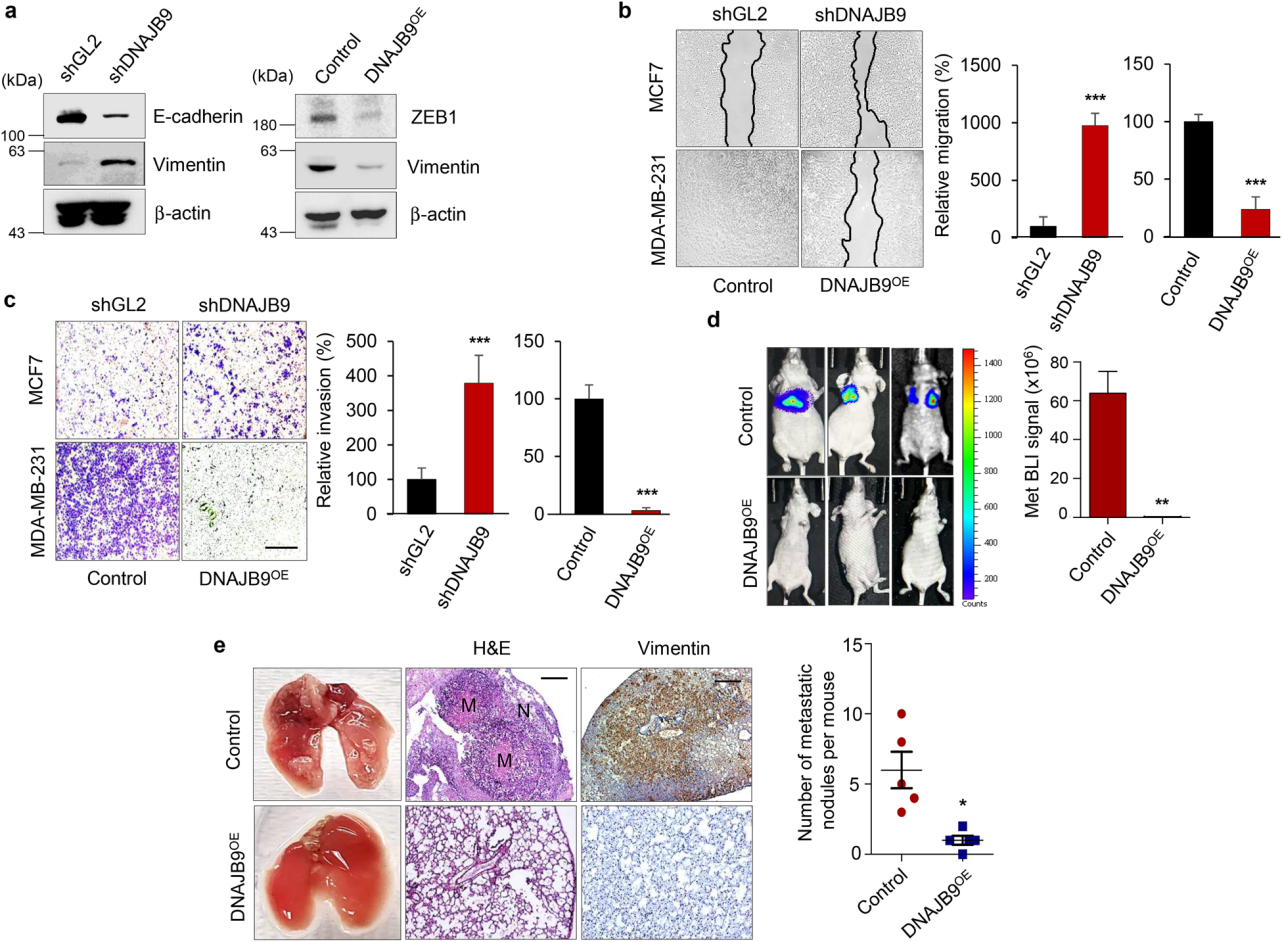
DNAJB9 suppresses the migration and metastasis of TNBC in vitro and in vivo. **a** Epithelial–mesenchymal transition markers (ZEB1, E-cadherin, and Vimentin) were validated in MCF7–shDNAJB9 and MDA-MB-231–DNAJB9-OE cells using western blotting. β-Actin was used as normalization controls. **b**, **c** To measure the metastatic potential of cancer cells, wound healing (**b**) and transwell cell invasion assay (**c**) were performed in MCF7–shDNAJB9 and MDA-MB-231–DNAJB9-OE cells. Representative images are shown in the left panel. The quantitative results are shown in the right panel. Scale bar, 500 μm. **d** MDA-MB-231–DNAJB9-OE or control cells (1 × 10^6^) were intravenously injected into 6-week-old female immunodeficient mice. The representative bioluminescence images of lung metastasis and the quantification of the bioluminescence intensity. **e** Representative images of metastatic lung tumors, hematoxylin and eosin (H&E), and Vimentin are shown. The number of lung tumors derived from MDA-MB-231–DNAJB9-OE or control cells was quantitatively analyzed. Scale bar, 200 μm. Results are presented as mean ± standard deviation from three independent experiments. *p < 0.05; **p < 0.01; ***p < 0.001.

To evaluate whether DNAJB9 could functionally regulate metastasis of breast cancer cells, we established the DNAJB9-KD or DNAJB9-OE MCF7 or MDA-MB-231 cells, respectively. The expression level of DNAJB9 in stable cell lines was evaluated using western blotting and qRT–PCR (Supplementary Fig. S3a, b). Interestingly, the KD of DNAJB9 in MCF7 cells displayed a typical spindle-shaped morphology of mesenchymal cells. However, the OE of DNAJB9 in MDA-MB-231 cells showed a more spherical morphology typical of epithelial cells (Supplementary Fig. S3c). Cancer cells possess a high level of phenotypic plasticity or the ability to transition between EMT and MET states. During the EMT state, epithelial cells loosen cell–cell adhesion and their adhesive properties and acquire mesenchymal phenotypes, such as migration and invasion^31,32^. Therefore, whether DNAJB9 is involved in the EMT process in breast cancer was confirmed. Western blotting showed that DNAJB9 KD in MCF7 cells decreased the levels of the epithelial marker, E-cadherin (Fig. 2a). Meanwhile, the mesenchymal marker, Vimentin, was increased in MCF7-shDNAJB9 cells compared with parental cells. Alternatively, the OE of DNAJB9 in MDA-MB-231 cells reduced the level of the mesenchymal markers, ZEB1 and Vimentin, compared with control cells (Fig. 2a). Furthermore, to verify the effect of DNAJB9 on nuclear translocation of ZEB1, we performed immunocytochemistry. As a result, OE of DNAJB9 decreased the nuclear accumulation of ZEB1, and conversely, KD of DNAJB9 led to increased nuclear translocation of ZEB1 (Supplementary Fig. S3d). Consistent with the morphological changes, the migration and invasion abilities of DNAJB9-KD MCF7 cells were dramatically increased compared with those of parental cells (Fig. 2b, c). In contrast, DNAJB9 OE in MDA-MB-231 cells had a significantly reduced ability to migrate and invade compared with control cells. However, the modulation of DNAJB9 expression did not affect the proliferative capacity of breast cancer cells (Supplementary Fig. S3e).

To further investigate the effect of DNAJB9 on the metastatic capacity of breast cancer cells in vivo, we intravenously injected DNAJB9-overexpressing MDA-MB-231 cells expressing the luciferase reporter into the tail vein of immunodeficient mice as an experimental model to assess metastatic colonization. Consistent with the in vitro results, bioluminescence imaging revealed that DNAJB9 OE significantly inhibited the metastatic colonization of breast cancer into the lung (Fig. 2d). Additionally, human Vimentin-positive staining in the DNAJB9-overexpressing MDA-MB-231 group was decreased compared to the control group (Fig. 2e). Collectively, these results indicate that DNAJB9 acts as a novel negative regulator of EMT and metastasis of breast cancer cells.

### DNAJB9 promotes ZEB1 degradation in breast cancer cells

**Fig. 3:**
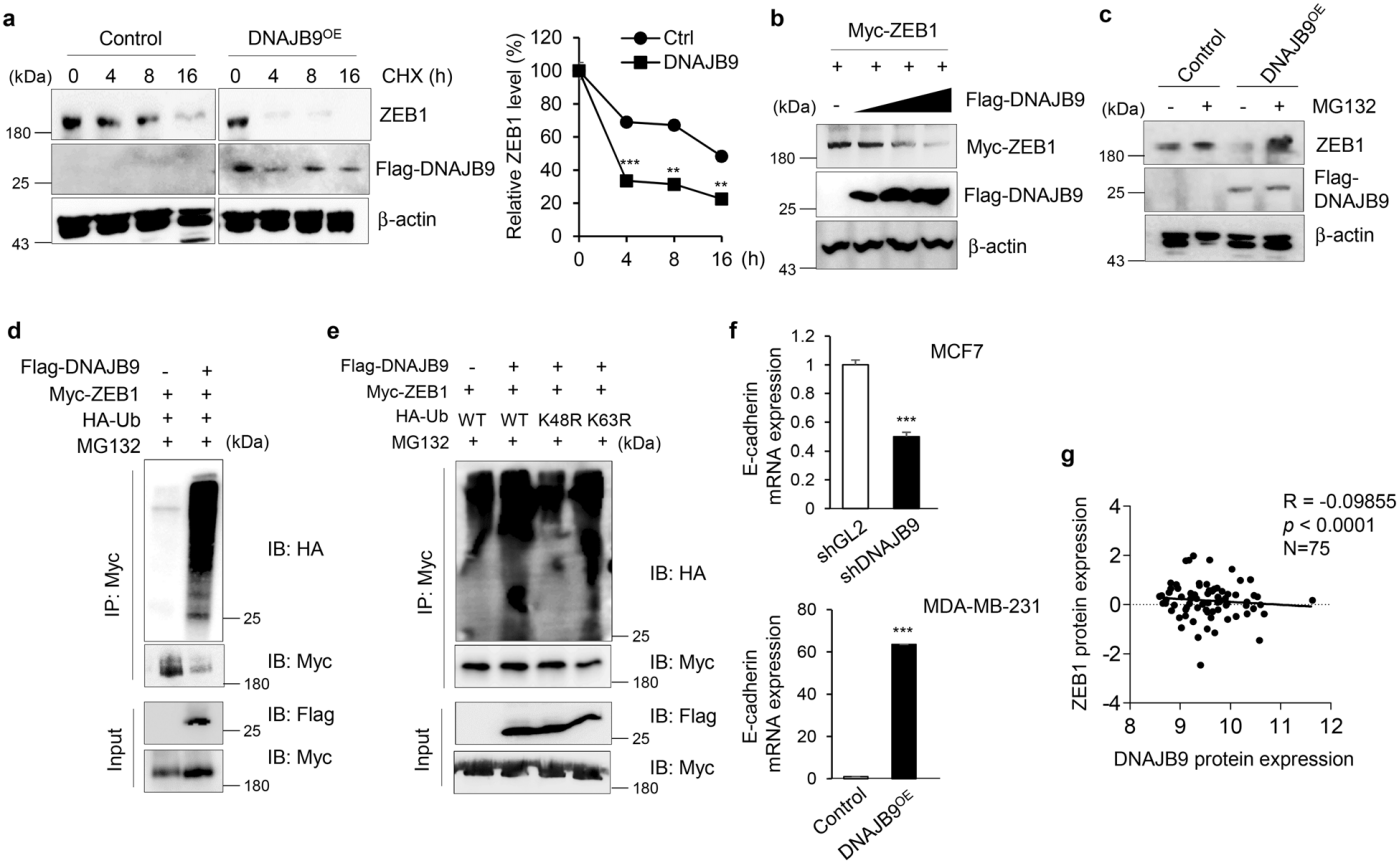
DNAJB9 promotes ZEB1 degradation via a proteasomal pathway. **a** Western blotting. Stability of ZEB1 was detected using western blotting of MDA-MB-231–DNAJB9-OE or control cells in 10 μM cycloheximide for the indicated times (left panel). The band intensity of ZEB1 at each time point was normalized compared with that of β-actin and converted into percentages using 100% as the value of zero time point (right panel). **b** To determine the inhibitory effect of DNAJB9 on ZEB1, increasing amounts of DNAJB9 constructs were transfected into HEK293 cells. The level of ZEB1 was detected using western blotting. **c** To check the proteasome-mediated degradation of ZEB1 by DNAJB9, MDA-MB-231 cells were incubated with MG132 (10 μM) for 16 h, and the level of ZEB1 was examined using western blotting. **d** HEK293T cells were transiently co-transfected with the control vector, Flag-DNAJB9, and with Myc-ZEB1 or HA-ubiquitin as indicated. At 36 h post-transfection, the cells were incubated with MG132 for 6 h and then subjected to ubiquitination assay. In all, 10% of cell lysates were used for the analysis of input. **e** To determine the DNAJB9-mediated ubiquitination type of ZEB1, DNAJB9 and ZEB1 constructs were transfected into HEK293T cells with various ubiquitin plasmids. Ubiquitinated ZEB1 was detected with anti-HA antibody from immunoprecipitated ZEB1 protein. **f** The expression level of E-cadherin gene was detected using qRT–PCR in DNAJB9-modulated MCF7 and MDA-MB-231 cells. Cyclophilin was used as normalization controls. **g** The Pearson correlation between DNAJB9 and ZEB1 protein expression in 75 breast cancer tissues from The Cancer Genome Atlas database. The Pearson correlation coefficient (*R*) and *p* value (*p*) are reported. Results are presented as the mean ± standard deviation from three independent experiments. **p < 0.01; ***p < 0.001.

ZEB1 is a potent negative regulator of E-cadherin gene by interacting with two E-box sites in promoter regions allowing the invasion and metastasis of cancer^33,34^. To validate whether DNAJB9 regulates the expression of E-cadherin by modulating ZEB1, we performed ZEB1 protein stability assay using CHX. As a result, the OE of DNAJB9 in MDA-MB-231 cells considerably shortened the half-life of ZEB1 protein compared with control cells (Fig. 3a). In addition, ectopic expression of DNAJB9 dose-dependently decreased ZEB1 expression (Fig. 3b). Because ubiquitin-mediated proteasomal degradation is a critical mechanism that regulates protein stability, we used MG132, a proteasome inhibitor, to investigate whether ZEB1 was regulated through the ubiquitin–proteasome pathway. The results showed that DNAJB9-induced degradation of ZEB1 was markedly abolished by MG132 treatment (Fig. 3c), indicating that DNAJB9 accelerates ZEB1 degradation through the ubiquitin–proteasome pathway. To examine the effect of DNAJB9 on the ubiquitination of ZEB1, we co-transfected DNAJB9 with ZEB1 and ubiquitin constructs into HEK293 cells. As shown in Fig. 3d, the ectopic expression of DANJB9 dramatically increased the ubiquitination of ZEB1.

As known, K48-linked polyubiquitin chains are associated with directing substrates for proteasomal degradation, whereas K63-linked polyubiquitin is associated with cell signaling events in intracellular signaling, DNA repair, and cytokine signaling^35–37^. To understand which type of ubiquitination occurs on ZEB1 using DNAJB9, we used ubiquitin mutant constructs in which the lysine residue at positions 48 and 63 was replaced with arginine (K48R and K63R). Ubiquitination assay revealed that ZEB1 was ubiquitinated by DNAJB9 via K48-linked chains, but not K63-linked chains (Fig. 3e). Consistent with the downregulation of negative regulators, E-cadherin promoter activity markedly increased in the presence of DNAJB9 (Supplementary Fig. S4a). Consistently, the expression level of E-cadherin mRNA was positively correlated with that of DNAJB9 (Fig. 3f). Intriguingly, we discovered a negative correlation between ZEB1 and DNAJB9 protein levels in 75 breast cancer tissue samples (*R* = −0.09855; *p* < 0.0001; Fig. 3g). However, the level of DNAJB9 mRNA was positively correlated with that of E-cadherin mRNA in 455 breast cancer samples (*R* = 0.092; *p* < 0.05; Supplementary Fig. S4b). These data outlined that DNAJB9 suppresses the metastasis of breast cancer by promoting ZEB1 degradation and E-cadherin expression.

### DNAJB9 promotes ZEB1 degradation through FBXO45-dependent ubiquitination

**Fig. 4:**
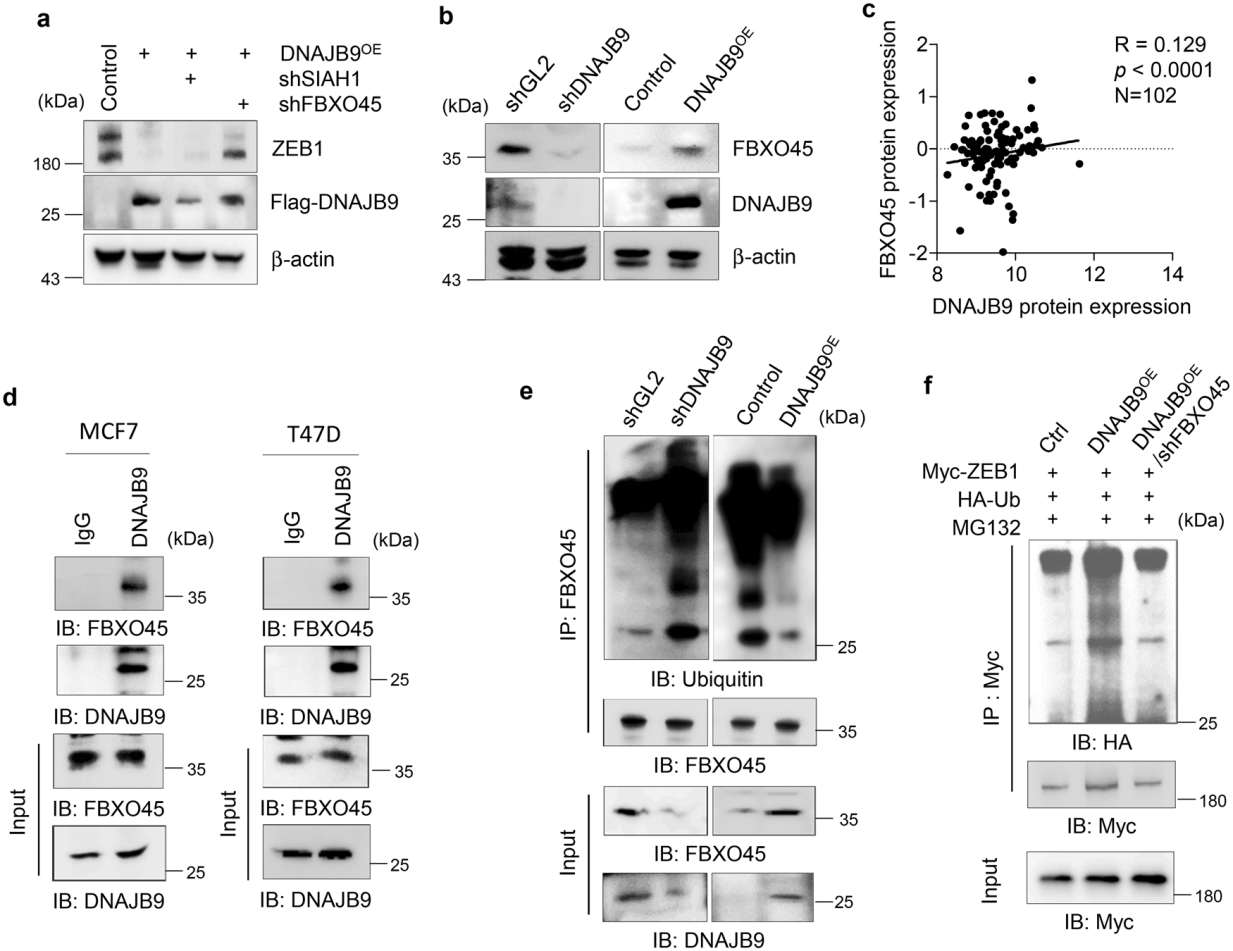
DNAJB9 regulates ZEB1 through FBXO45-mediated ubiquitination. **a** To identify the target for ZEB1 degradation, shRNAs against SIAH1 or FBXO45 were introduced into MDA-MB-231 cells. Then the expression level of ZEB1 proteins was checked using western blotting. **b** The expression levels of FBXO45 proteins were checked using western blotting in MCF7–shDNAJB9 and MDA-MB-231–DNAJB9-OE cells. **c** The Pearson correlation between DNAJB9 and FBXO45 protein expression in 102 breast cancer tissues from The Cancer Genome Atlas database. The Pearson correlation coefficient (*R*) and *p* value (*p*) are reported. **d** To confirm the interaction between DNAJB9 and FBXO45, MCF7 and T47D cell lysates were subjected to immunoprecipitation with control IgG or anti-DNAJB9 antibodies. The immunoprecipitates were detected using the indicated antibodies. **e** To confirm the inhibitory effect of DNAJB9 on FBXO45 ubiquitination, MCF7–shDNAJB9 and MDA-MB-231–DNAJB9-OE cell lysates were subjected to immunoprecipitation with an anti-FBXO45 antibody. Then the immunoprecipitated FBXO45 was detected with an anti-ubiquitin antibody. **f** To validate the FBXO45-mediated ubiquitination of ZEB1, indicated cells were transfected using ZEB1 and ubiquitin constructs. Then the immunoprecipitated ZEB1 was detected with an anti-HA antibody. Results are presented as mean ± standard deviation from three independent experiments.

Accumulating evidence has elucidated that chaperone-interacting ubiquitin ligases induce the degradation of chaperone client proteins^38–40^. Therefore, we assumed that DNAJB9 degrades ZEB1 depending on ubiquitin ligases. SIAH1 and FBXO45 are well-known ubiquitin ligases for ZEB1, leading to the inhibition of EMT and metastasis in human malignancies^41,42^. To confirm the possibility whether DNAJB9 induces proteasomal degradation of ZEB1 through E3 ligases, SIAH1, or FBXO45, we knocked down SIAH1 or FBXO45 using shRNA in MDA-MB-231–DNAJB9-OE cells, respectively. The loss of FBXO45 in DNAJB9-OE cells significantly increased ZEB1 proteins similar to control cells (Fig. 4a). However, no significant change in ZEB1 expression was observed in DNAJB9-OE–shSIAH1 cells (Fig. 4a). These results indicated that DNAJB9 modulates the level of ZEB1 through a FBXO45-dependent pathway.

The regulation of client proteins by Hsp chaperones is involved in critical cellular processes controlling the folding, transport, degradation, and assembly of proteins. Numerous recent studies have elucidated that the Hsp chaperone machinery interacts with more than a hundred substrate proteins, including ubiquitin ligase, and regulates their activity^43,44^. Thus, we speculated that FBXO45 is a client protein of DNAJB9 to target ZEB1. Then we found that the protein level of FBXO45 was reduced in MCF7–DNAJB9-KD cells, whereas that was increased in MDA-MB-231–DNAJB9-OE cells (Fig. 4b). However, the level of FBXO45 mRNA was not affected by the modulation of DNAJB9 expression (Supplementary Fig. S5a). Moreover, the protein level of FBXO45 was significantly elevated in luminal cells compared with that in TNBC cells (Supplementary Fig. S5b). Then we performed IHC staining in 26 cases of paraffin-embedded human breast tumor specimens and found that FBXO45 was significantly higher in luminal tissues than those in TNBC tissues (Supplementary Fig. S5c). Furthermore, data from LinkedOmics showed that the protein expression of FBXO45 was positively correlated (*R* = 0.129; *p* < 0.0001) with DNAJB9 protein expression in 102 breast cancer tissue samples (Fig. 4c). To determine whether FBXO45 is a client protein of DNAJB9, we performed reciprocal co-immunoprecipitation assays using in vitro and endogenous systems. As shown in Fig. 4d, FBXO45 strongly interacts with DNAJB9 at endogenous conditions. In addition, the interaction between FBXO45 and DNAJB9 was confirmed using a vice versa transient OE system (Supplementary Fig. S5d, e). Altogether, these results demonstrate that DNAJB9 governs FBXO45 expression through their interaction in breast cancer. In addition, FBXO, a member of the F-box protein family, serves as a substrate receptor of the SCF (Skp1-Cul1-F-box) E3 ligase complex^45^. Moreover, the F-box domain of FBXO proteins affected self-ubiquitination and contributed to its destabilization^46^. Therefore, we further examined whether DNAJB9 affects the self-ubiquitination of FBXO45. As a result, DNAJB9 significantly inhibited FBXO45 self-ubiquitination in vitro (Supplementary Fig. S5f) and at endogenous conditions (Fig. 4e).

Next, we determined whether DNAJB9 induces the ubiquitin-dependent degradation of ZEB1 via FBXO45. As expected, the ectopic expression of FBXO45 dramatically increased the ubiquitination of ZEB1 (Supplementary Fig. S5g). Our data further showed that DNAJB9 induced the ubiquitination of ZEB1, whereas the KD of FBXO45 significantly reduced the ubiquitination of ZEB1 (Fig. 4f). These results indicated that DNAJB9 induced the ubiquitin-dependent degradation of ZEB1 through FBXO45.

### DNAJB9 suppresses the metastasis of TNBC through FBXO45

**Fig. 5:**
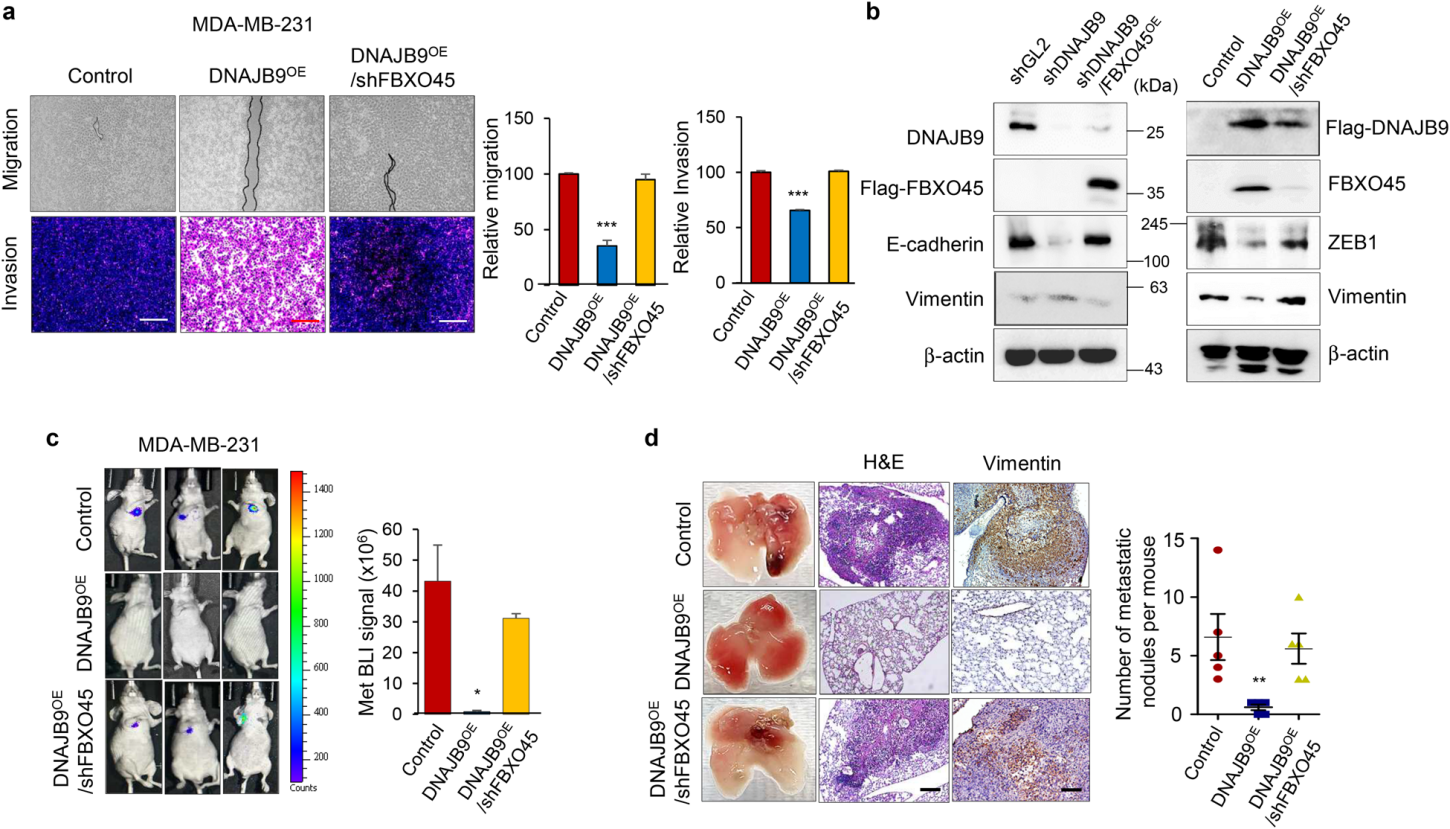
DNAJB9 suppresses the metastasis of TNBC through FBXO45. **a** To measure the effect of DNAJB9 and FBXO45 on cell migration, cells were plated into six-well plates and wounded using pipette tips. After 24 h, the migrated cells were captured using a microscope and quantified using ImageJ. To measure the effect of DNAJB9 and FBXO45 on cell invasion, cells were plated into 8-μm inserts. After 24 h, the invaded cells were stained with crystal violet, and signal intensity was measured using ImageJ. Results are presented as mean ± standard deviation from three independent experiments. Scale bar, 500 μm. **b** Epithelial–mesenchymal transition markers were validated in MCF7-shDNAJB9–FBXO45-OE and MDA-MB-231–DNAJB9-OE–shFBXO45 cells using western blotting. A β-actin band was used as normalization control. **c** Representative images (left) and bioluminescence imaging signals (right) of lung metastasis after intravenous injection of the control, DNAJB9 OE or DNAJB9 OE/shFBXO45 cell lines (1 × 10^6^) into 6-week-old female nude mice (5 mice in each group) 10 weeks post injection. The luciferase activity was detected using the IVIS Spectrum after injection of luciferin. **d** Representative images of metastatic lung tumors, hematoxylin and eosin (H&E), and human Vimentin immunohistochemical staining are shown (left), with the number of lung tumors derived from the indicated cell lines that were quantitatively analyzed (right). Scale bar, 200 μm. Results are presented as mean ± standard deviation from three independent experiments. *p < 0.05; **p < 0.01; ***p < 0.001.

Because DNAJB9 modulated the FBXO45-mediated negative regulation of ZEB1, we further investigated whether DNAJB9 and FBXO45 affect the metastasis of breast cancer. The expression of FBXO45 restored the suppressive activity of luminal breast cells in terms of migration and invasion (Supplementary Fig. S6). In contrast, the inhibitory ability of DNAJB9 on migration and invasion were significantly reversed by the loss of FBXO45 in TNBC cells (Fig. 5a). Consistent with the migration and invasion data, EMT markers were differentially expressed in FBXO45-modulated cells (Fig. 5b). The reduction of E-cadherin and increase of Vimentin by DNAJB9 loss in epithelial cells were reversed by restoring FBXO45. However, the suppression of mesenchymal markers by DNAJB9 was abolished by the loss of FBXO45 in TNBC cells (Fig. 5b). In agreement with these results, in vivo metastasis model showed that the suppression of FBXO45 could reverse the anti-metastatic effects of DNAJB9 and increase metastatic colonization into the lungs (Fig. 5c, d). Altogether, our results demonstrate that the novel DNAJB9–FBXO45 axis acts as an important negative regulator of metastasis of TNBC.

## Discussion

**Fig. 6:**
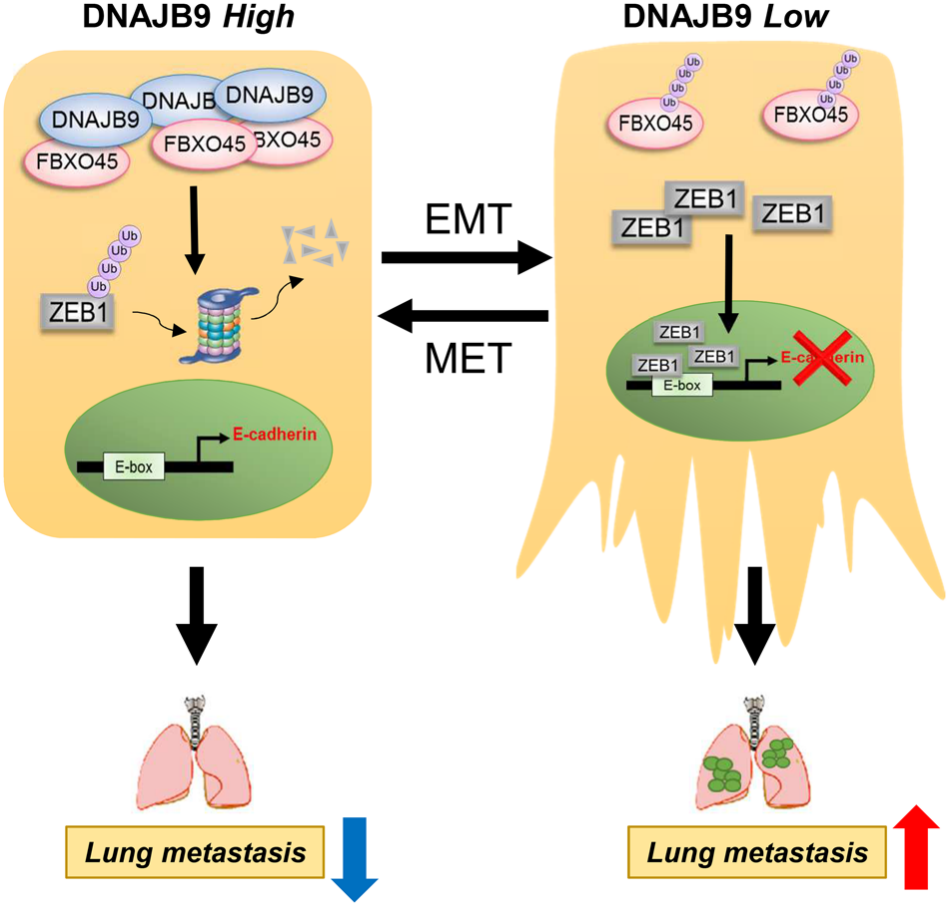
A proposed model for the role of DNAJB9 in breast cancer metastasis. With a high level of DNAJB9, FBXO45 was stabilized by interacting with DNAJB9 and induced the proteasomal degradation of ZEB1 through K48-mediated ubiquitination in non-TNBC cells. As a result, the epithelial–mesenchymal transition (EMT) process was suppressed by inducing the transcription of epithelial cell markers, followed by the inhibition of metastasis into the lungs. However, the loss or low level of DNAJB9 allowed the expression of ZEB1 to inhibit the transcription of E-cadherin and resulted in EMT progression. Finally, TNBC cells can metastasize into the lungs and threaten patient survival.

In this study, we propose that the novel DNAJB9–FBXO45 signaling axis synergistically suppresses the metastatic progression of breast cancer, and the modulation of this network is an effective therapeutic strategy against metastatic TNBC (Fig. 6). Using human breast cancer cell lines, preclinical models, and clinical samples, we showed that loss of DNAJB9 expression is associated with increased tumor aggressiveness and shorter OS and DMFS of patients. The restoration of DNAJB9 in aggressive TNBC cells profoundly suppressed both in vitro invasion and in vivo metastasis. Mechanistically, we identified that DNAJB9 could stabilize FBXO45 proteins, inducing ZEB1 ubiquitination and degradation. Importantly, given that DNAJB9 and FBXO45 are tightly correlated in patients with breast cancer, our findings highlight DNAJB9 as a potential biomarker for predicting patient survival and a novel therapeutic target for treating patients with breast cancer.

Heat shock proteins (HSPs) are known as a molecular chaperone, which is conserved in all mammalian cells and functions to maintain cellular homeostasis in response to stress such as hypoxia, high temperature, and chemical agents. However, HSPs are also expressed in cells even under normal conditions. Recently, numerous studies have revealed that HSPs are a promising target for anti-cancer therapy. In particular, HSP70 and HSP90 activity in multiple cellular pathways has been widely studied and has a direct relevance to human cancer pathogenesis. Accordingly, several Hsp90 inhibitors have been developed and evaluated in cancer clinical trials^47–49^. Moreover, HSP40 inhibitors, KNK437, and BMS-690514, effectively suppress cancer progression and metastasis in colorectal cancer and non-small cell lung carcinoma^50,51^. Because no drugs or chemicals enhance the activity of HSP protein for anti-cancer purposes, further study is needed to develop a therapeutic strategy for DNAJB9-dependent drugs against TNBC metastasis.

This study presents the importance of DNAJB9 as a promising candidate for suppressing metastasis of TNBC. First, we observed that DNAJB9 expression was significantly suppressed in metastasized breast cancer samples compared to localized breast cancer. Interestingly, the expression of other DNAJ–HSP40 proteins (DNAJB4 and DNAJB6) that are known as tumor suppressors was increased in metastatic TNBC cell lines (Supplementary Fig. 2a). These discrepancies may originate from the differences in splice variants of DNAJ-HSP40 proteins. Previous studies suggested that various splice variants of HSPs exhibit distinct expression profiles and functions according to the breast cancer subtypes based on comprehensive transcriptomic analysis^29,52^. Second, a series of functional analyses using in vitro and in vivo experiments suggested that DNAJB9 could function as a tumor suppressor by inhibiting the migration and invasion of breast cancer cells and suppressing metastasis in xenograft models. Finally, we also characterized the mechanism by which DNAJB9 regulates breast cancer cell invasion and metastasis by regulating ZEB1 and the EMT process. Based on these results, we will develop a screening system to identify the chemicals that induce the expression of DNAJB9 to suppress the metastasis of TNBC.

Accumulating evidence shows that FBXO45 has diverse roles in tumorigenesis and tumor progression^53^. FBXO45 is highly expressed in several human cancers, such as squamous cell lung carcinoma and pancreatic cancer, and correlated with shortened OS and poorer outcomes^54,55^. However, other studies have demonstrated that FBXO45 might have an inverse role in several types of human malignancies. For instance, patients with gastric cancer with low FBXO45 expression exhibited poorer survival and prognoses than those with high FBXO45 expression^56^. Moreover, FBXO45 inhibited cancer development and metastasis by targeting EMT-inducing transcription factors, including SNAI1/2, TWIST1/2, and ZEB1/2, in various cancer cells^36,57^. In agreement with previous studies, we showed that FBXO45 functions as a tumor suppressor, particularly for more aggressive breast cancer subtypes, indicating that the anti-metastatic effects of FBXO45 are strikingly cancer-type specific.

A combination of experimental, clinical, and bioinformatics analyses has revealed the biological significance of DNAJB9–FBXO45 regulation in breast cancer progression and metastasis. Our results demonstrate that DNAJB9 is an indispensable regulator for FBXO45 and that the DNAJB9–FBXO45–ZEB1 signaling axis may not only serve as a prognostic marker but also provide opportunities for developing therapeutic interventions in metastatic breast cancers.

## Supplementary information

Supplementary document

Supplementary figure
