# Supplementary material for: DNAJB9 suppresses the metastasis of triple-negative breast cancer by promoting FBXO45-mediated degradation of ZEB1
Source: Cell Death Dis. 2021 May 8;12(5):461. doi: 10.1038/s41419-021-03757-x (PMC8106677; doi:10.1038/s41419-021-03757-x)
Supplement: Supplementary file 1 — Supplementary document [file 41419_2021_3757_MOESM1_ESM.docx]

**Supplementary Figure Legends**

**Supplementary Fig. S1 Low expression of DNAJB9 is associated with poor clinical outcomes. a** DNAJB9 mRNA levels across cancer types were determined using the GENT database. Breast normal and cancer samples were marked with a red box. **b** Genetic variants associating with OS (N = 1,402) and DMFS (N = 1,746) in breast cancer patients. All of the covariates that displayed significant associations with OS and DMFS were introduced in the Cox proportional hazards regression model.

**Supplementary Fig. S2 The expressions of DNAJB4 and DNAJB6 are independent of breast cancer patient survival. a** Scatter dot plots of DNAJB4 and DNAJB6 mRNA levels in a panel of 52 breast cancer cell lines using public microarray datasets. **b** Kaplan–Meier analysis of OS and DMFS of breast cancer patients. Patients with tumors expressing DNAJB4 and DNAJB6 at levels higher than the mean value are labeled in red, whereas those with tumors with gene expression levels below the mean are shown in blue. *P* values were calculated by the log-rank Mantel–Cox test. ***p* < 0.01.

**Supplementary Fig. S3 DNAJB9 induces the morphological changes of breast cancer cells. a** To inhibit the expression of DNAJB9, lentiviral shRNA against DNAJB9 was introduced into MCF7 cell. Infected cell was selected with puromycin and lysed to extract the total RNA and protein. Knockdown efficiency of DNAJB9 was assessed by qRT–PCR (left) and Western blotting (right). **b** To generate the DNAJB9-OE cell lines, MDA-MB-231 cells were infected with the lentiviral pCAG–Flag–DNAJB9 system. After 48 h incubation, infection efficiency was assessed using qRT–PCR (left) and Western blotting (right). Cyclophilin and β-actin were used as normalization controls for RNA and protein, respectively. **c** Representative photographs of DNAJB9-KD MCF7 cells (shDNAJB9) or control cells (shGL2). DNAJB9 OE in MDA-MB-231 cells (DNAJB9-OE) or corresponding control cells (Ctrl) were also photographed in same scale. Scale bar = 50 μm. **d** To determine the effect of DNAJB9 on nuclear localization of ZEB1, the immunocytochemistry was performed with ZEB1-specific antibody in DNAJB9-modulated MCF7 and MDA-MB-231 cells. The location of protein was detected with confocal fluorescence microscopy. The nuclei were visualized with DAPI staining (blue). Scale bar = 50 μm. **e** To confirm the effect of DNAJB9 on cell proliferation, MTT assay was performed with DNAJB9-modulated MCF7 and MDA-MB-231 cells. The data were compared with control cells. Results are presented as the mean ± SD from three independent experiments. ****p* < 0.001.

**Supplementary Fig. S4 DNAJB9 increases the transcription of E-cadherin gene.** **a** To validate the effects of DNAJB9 on E-cadherin transcription, MDA-MB-231–DNAJB9-OE cells were transfected using E-cadherin luciferase reporter construct. After 48 h incubation, luciferase activity was measured with a luminometer. The β-galactosidase was used as internal controls to monitor the transfection efficiency. The level of DNAJB9 protein was assessed using Western blotting (lower). β-actin was used as a normalization control. **b** Pearson correlation between DNAJB9 and E-cadherin mRNA expression in 455 breast cancer tissues from the GEO database (GSE133394). Pearson correlation coefficient (R) and *p* value (*P*) are reported. Results are presented as mean ± standard deviation from three independent experiments. ****p* < 0.001.

**Supplementary Fig. S5 FBXO45 regulates ZEB1 through DNAJB9-dependent ubiquitination in breast cancer.** **a** The expression levels of FBXO45 mRNAs were checked using qRT–PCR in MCF7–shDNAJB9 and MDA-MB-231–DNAJB9-OE cells. Cyclophilin was used as normalization controls. **b** The expression of FBXO45 protein was checked by Western blotting in luminal and TNBC cell lines. β-actin was detected as normalization controls. **c** Representative images of IHC staining of FBXO45 on paraffin-embedded human breast normal (n = 5), luminal (n = 9), and TNBC (n = 6) tissues (left panel). Images were captured and quantified using ImageJ from at least three fields (right panel). Scale bar = 100 μm. **d, e** To confirm the interaction of FBXO45 with DNAJB9, Flag-FBXO45 and Myc-DNAJB9 were co-transfected into HEK293T cells. The cell lysates were subjected to immunoprecipitation with anti-Myc or anti-Flag antibodies. The immunoprecipitates and input lysates were analyzed using Western blotting. **f** To confirm the inhibitory effect of DNAJB9 on self-ubiquitination of FBXO45, increasing amounts of DNAJB9, FBXO45, and ubiquitin constructs were transfected into HEK293 cells. Then, immunoprecipitated FBXO45 was detected with anti-HA antibody. **g** To validate the FBXO45-mediated ZEB1 ubiquitination, ZEB1 and FBXO45 constructs were transfected with ubiquitin DNA. Protein lysates were immunoprecipitated with anti-Myc antibody and ubiquitinated proteins were detected with anti-HA antibody. Results are presented as mean ± standard deviation from three independent experiments. ***p* < 0.01.

**Supplementary Fig. S6 DNAJB9–FBXO45 axis suppresses the migration and invasion of breast cancer.** To measure the effect of DNAJB9 and FBXO45 on cell migration, cells were plated into 6-well plates and wounded using pipette tips. After 24 h, migrated cells were captured using a microscope and quantified using ImageJ. Scale bar = 500 μm. To validate the effect of DNAJB9 and FBXO45 on cell invasion, cells were plated into 8-μm inserts. After 24 h, invaded cells were stained with crystal violet, and signal intensity was calculated using ImageJ. Results are presented as mean ± standard deviation from three independent experiments. ***p* < 0.01.

**Supplementary Tab. S1 The target sequences of shRNA used in this study**

| **Gene** | **Target sequences (5´ to 3´)** |
| --- | --- |
| shDNAJB9 | CTCAGATGCTAATAGACGAAA |
| shFBXO45 | ACTTCCAAAGGTCTGCTTATA |
| shSIAH1 | GCTTACCGACTTGAGCTAAAT |

**Supplementary Tab. S2 List of primer sequence used in this study**

| **Gene** | **Forward (5´ to 3´)** | **Reverse (5´ to 3´)** |
| --- | --- | --- |
| hDNAJB9 | AAGGCCTTTCACAAGTTGGC | ACGCTTCTTGGATCCAGTGTT |
| hEcad | ACAGCCCCGCCTTATGATT | TCGGAACCGCTTCCTTCA |
| hFBXO45 | AGTGCCAAGGTTATGTGG  CATTGCTG | AGAAAGCCACTGTCATCCGT  CCAAAG |
| hSIAH1 | TAAATGGTCATAGGCGACGA | GCAATGCTGGTGTCAAAGAC |
| hCyclo | TGCCATCGCCAAGGAGTAG | TGCACAGACGGTCACTCAAA |
