# Supplementary material for: DNAJB9 suppresses the metastasis of triple-negative breast cancer by promoting FBXO45-mediated degradation of ZEB1
Source: Cell Death Dis. 2021 May 8;12(5):461. doi: 10.1038/s41419-021-03757-x (PMC8106677; doi:10.1038/s41419-021-03757-x)
Supplement: Supplementary file 2 — Supplementary figure [file 41419_2021_3757_MOESM2_ESM.pptx]

## Slide 1
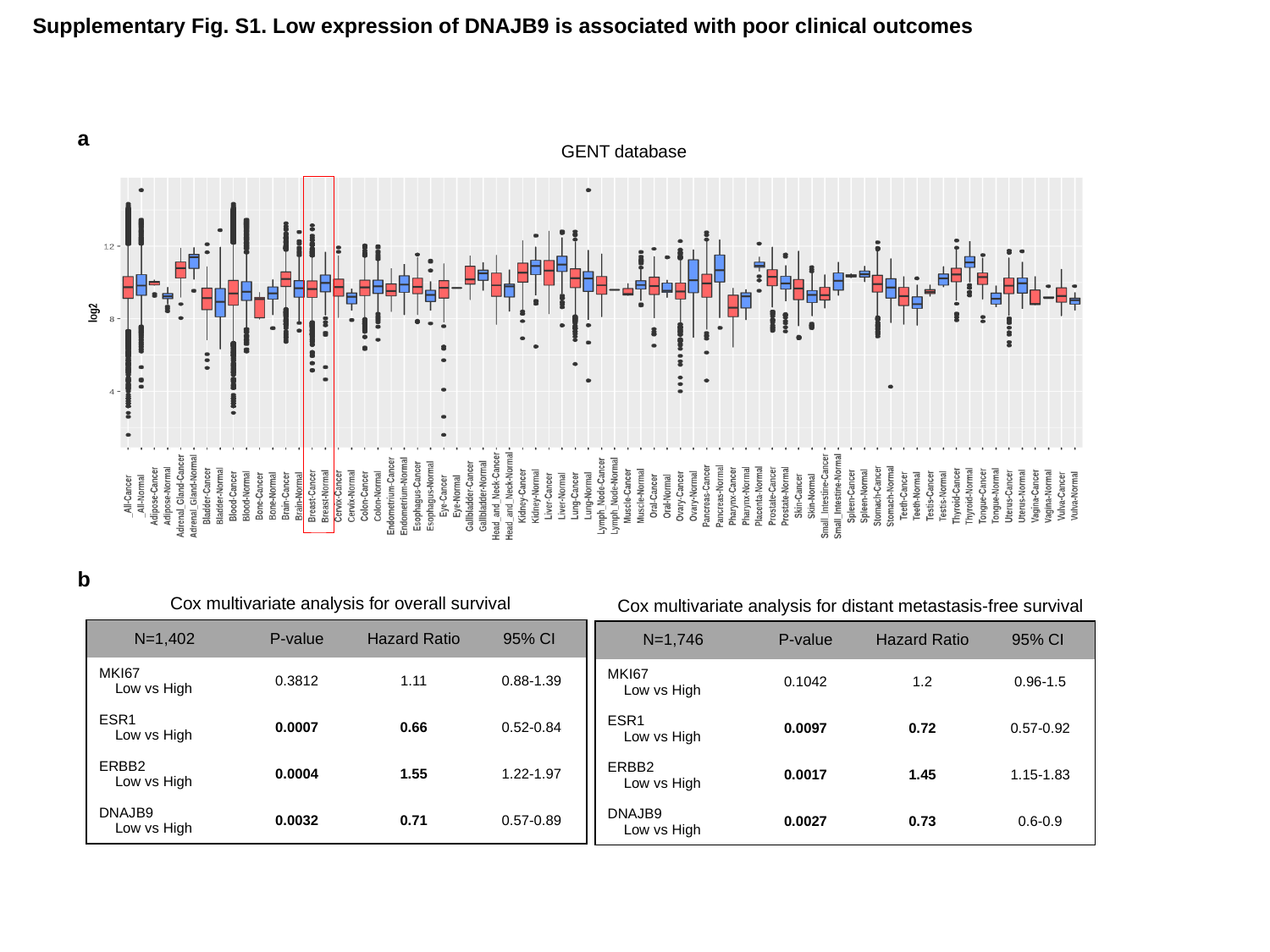

Supplementary Fig. S1. Low expression of DNAJB9 is associated with poor clinical outcomes
a
GENT database
b
Cox multivariate analysis for overall survival
Cox multivariate analysis for distant metastasis-free survival
| N=1,402 | P-value | Hazard Ratio | 95% CI |
| --- | --- | --- | --- |
| MKI67 Low vs High | 0.3812 | 1.11 | 0.88-1.39 |
| ESR1 Low vs High | 0.0007 | 0.66 | 0.52-0.84 |
| ERBB2 Low vs High | 0.0004 | 1.55 | 1.22-1.97 |
| DNAJB9 Low vs High | 0.0032 | 0.71 | 0.57-0.89 |
| N=1,746 | P-value | Hazard Ratio | 95% CI |
| --- | --- | --- | --- |
| MKI67 Low vs High | 0.1042 | 1.2 | 0.96-1.5 |
| ESR1 Low vs High | 0.0097 | 0.72 | 0.57-0.92 |
| ERBB2 Low vs High | 0.0017 | 1.45 | 1.15-1.83 |
| DNAJB9 Low vs High | 0.0027 | 0.73 | 0.6-0.9 |

## Slide 2
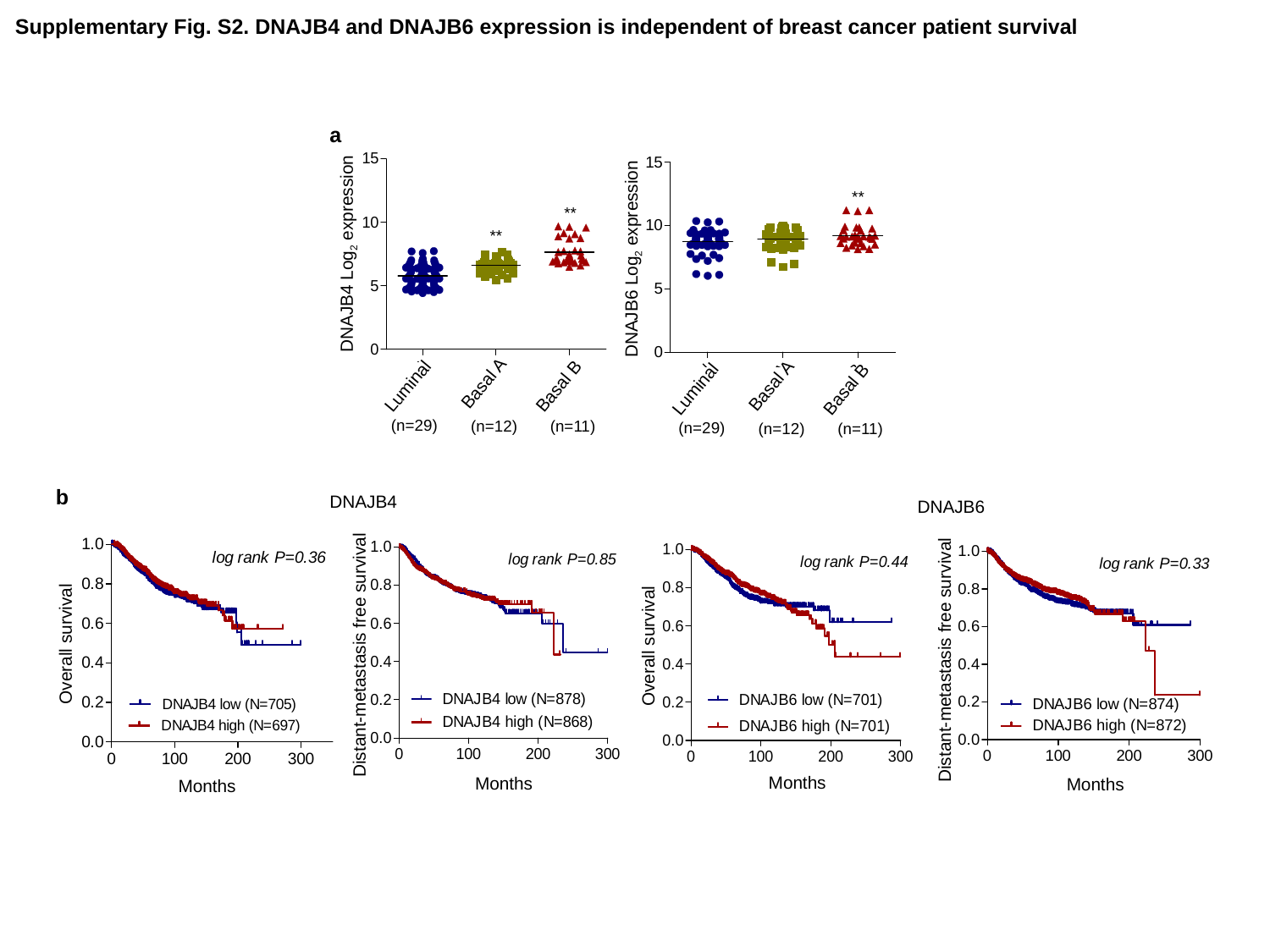

Supplementary Fig. S2. DNAJB4 and DNAJB6 expression is independent of breast cancer patient survival
a
DNAJB4 Log2 expression
Basal A
Luminal
Basal B
(n=29)
(n=12)
(n=11)
DNAJB6 Log2 expression
Basal A
Luminal
Basal B
(n=29)
(n=12)
(n=11)
**
**
**
b
DNAJB4
DNAJB6
Overall survival
Months
Overall survival
Distant-metastasis free survival
Distant-metastasis free survival
Months
Months
Months

## Slide 3
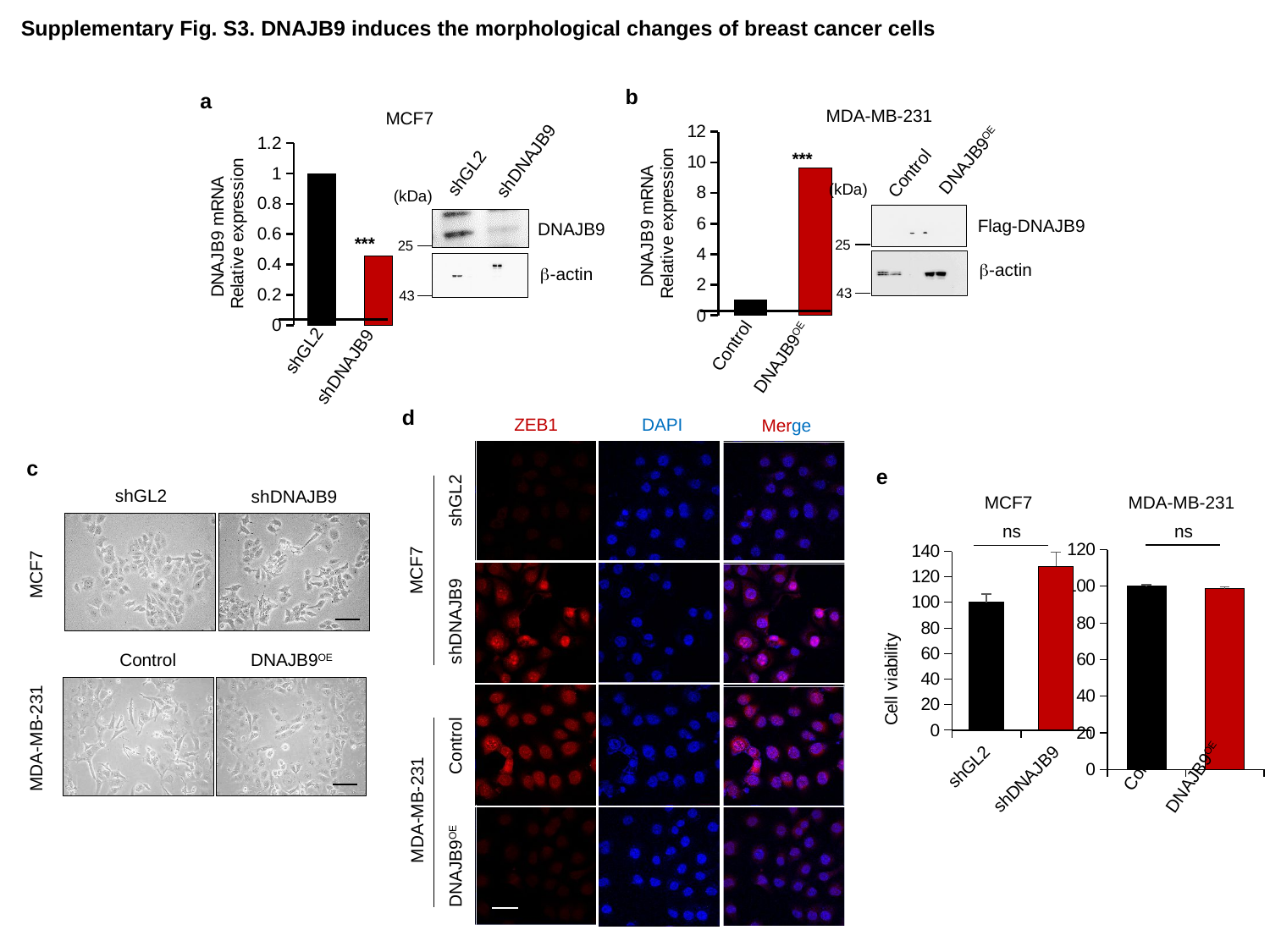

Supplementary Fig. S3. DNAJB9 induces the morphological changes of breast cancer cells
b
a
MDA-MB-231
DNAJB9OE
Control
Flag-DNAJB9
b-actin
MCF7
shDNAJB9
shGL2
DNAJB9
b-actin
### Chart
| Category | DNAJB9 |
|---|---|
| Cont | 1.0 |
| DNAJB9 OE | 9.61211939881973 |Control
DNAJB9OE
### Chart
| Category | |
|---|---|
| GL2 | 1.0 |
| shDNAJB9 | 0.4566404231820364 |***
shGL2
shDNAJB9
***
(kDa)
(kDa)
25
25
43
43
d
ZEB1
DAPI
Merge
c
e
shGL2
shDNAJB9
Control
DNAJB9OE
MCF7
shGL2
shDNAJB9
MCF7
MDA-MB-231
ns
ns
### Chart
| Category | |
|---|---|
| Ctrl | 100.0 |
| DNAJB9 | 98.63994273443093 |
### Chart
| Category | |
|---|---|
| shGL2 | 100.0 |
| shDNAJB9 | 128.21267412203255 |MCF7
Control
DNAJB9OE
MDA-MB-231
MDA-MB-231
Control
DNAJB9OE

## Slide 4
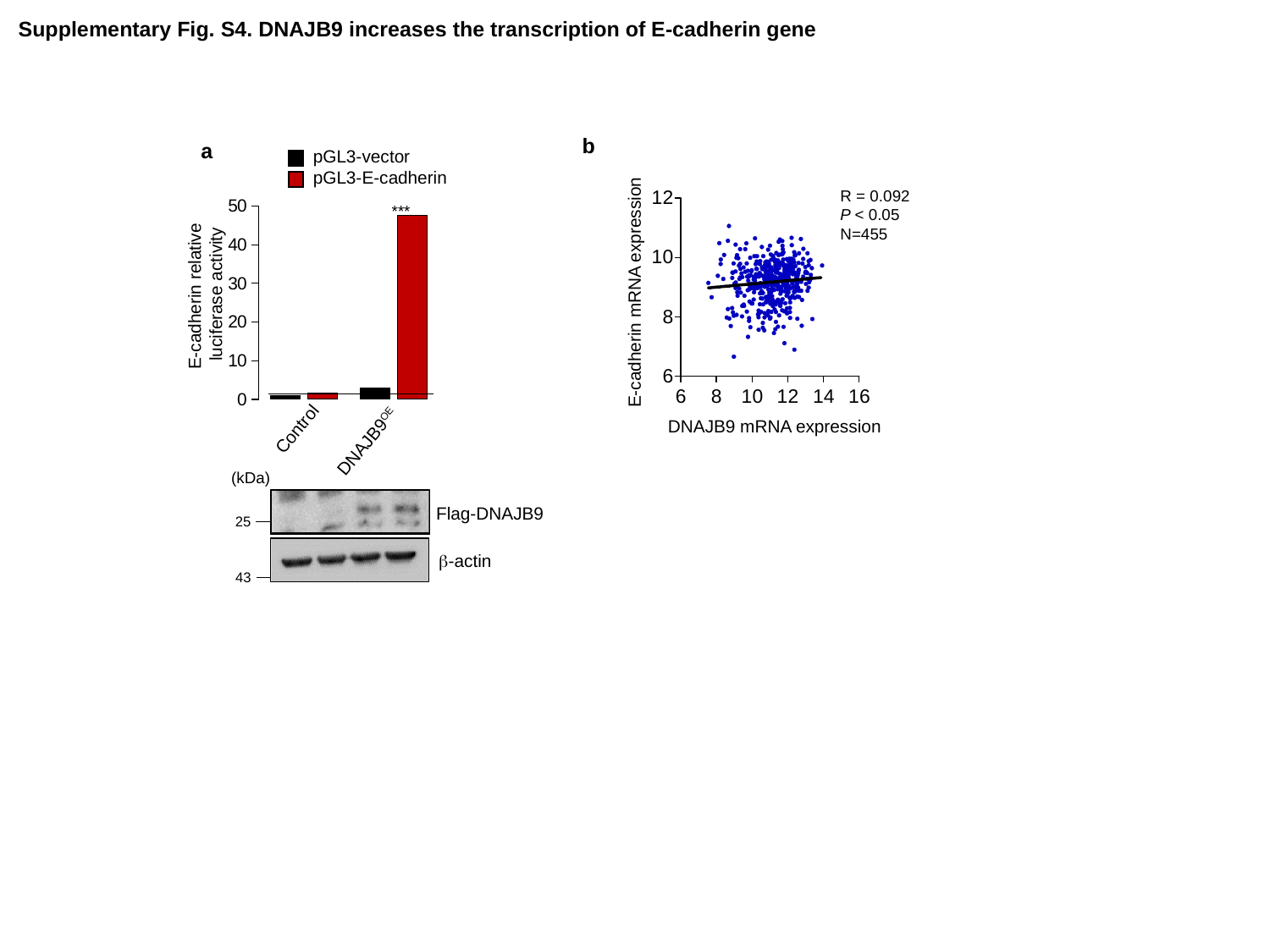

Supplementary Fig. S4. DNAJB9 increases the transcription of E-cadherin gene
b
a
pGL3-vector
pGL3-E-cadherin
R = 0.092
P < 0.05
N=455
DNAJB9 mRNA expression
### Chart
| Category | pGL3-Basic | pGL3-E-cad |
|---|---|---|
| Cont | 1.0 | 1.7110466197686895 |
| Flag-DNAJB9 | 2.9665224752593295 | 47.54728222248718 |***
E-cadherin relative
luciferase activity
E-cadherin mRNA expression
Control
DNAJB9OE
(kDa)
Flag-DNAJB9
b-actin
25
43

## Slide 5
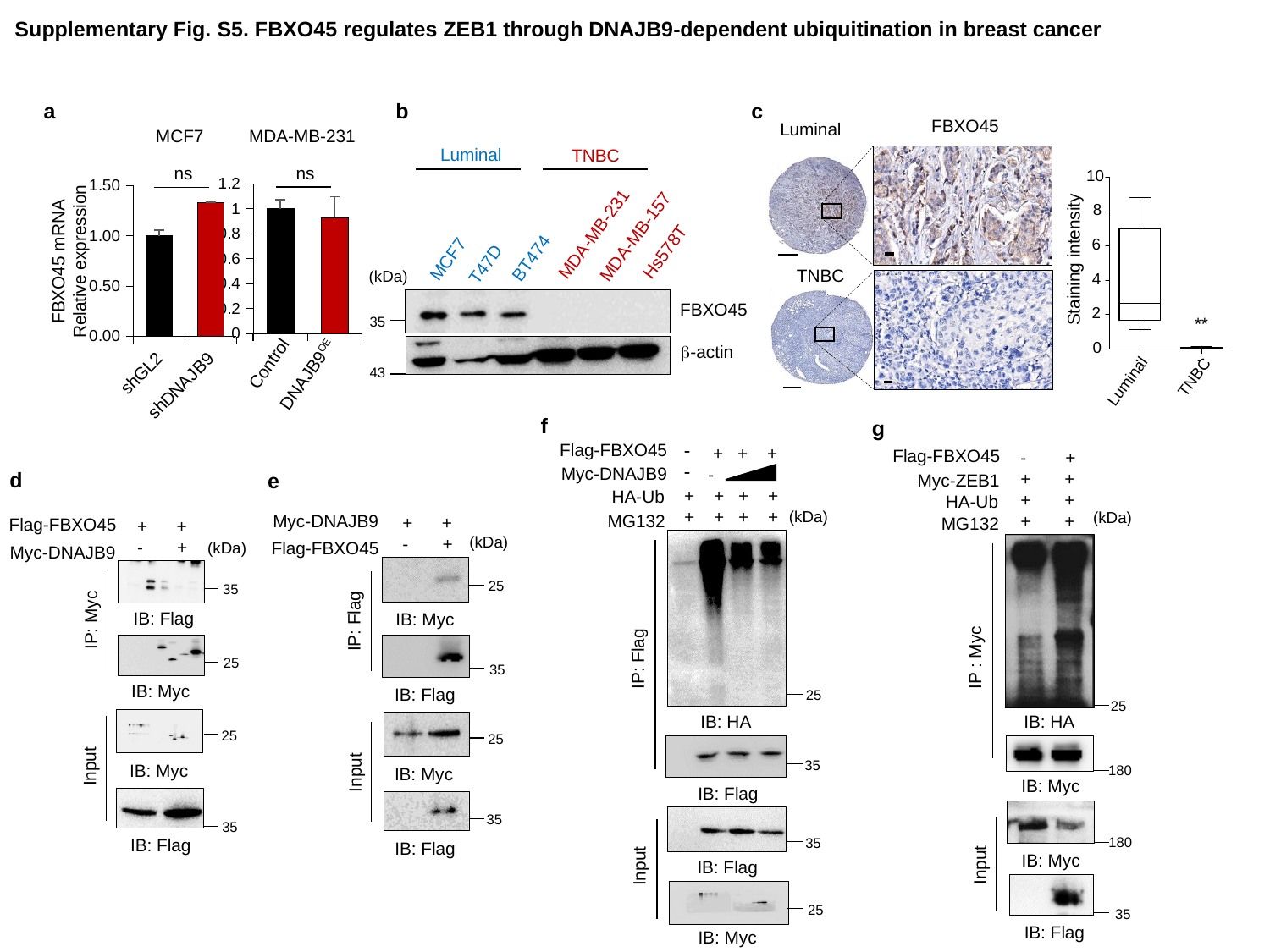

Supplementary Fig. S5. FBXO45 regulates ZEB1 through DNAJB9-dependent ubiquitination in breast cancer
a
b
c
FBXO45
Luminal
10
8
4
2
0
Staining intensity
TNBC
Luminal
6
**
TNBC
MCF7
MDA-MB-231
Luminal
TNBC
MDA-MB-231
MDA-MB-157
Hs578T
BT474
MCF7
T47D
FBXO45
β-actin
ns
ns
### Chart
| Category | |
|---|---|
| Ctrl | 1.0 |
| DNAJB9 | 0.9290327846147837 |
[unsupported chart]
FBXO45 mRNA
Relative expression
(kDa)
35
Control
DNAJB9OE
43
f
Flag-FBXO45
Myc-DNAJB9
HA-Ub
MG132
 + + +
- -
+ + + +
+ + + +
(kDa)
g
Flag-FBXO45
- +
+ +
+ +
+ +
d
e
Myc-ZEB1
HA-Ub
(kDa)
Myc-DNAJB9
+ +
- +
MG132
Flag-FBXO45
+ +
- +
(kDa)
Flag-FBXO45
(kDa)
Myc-DNAJB9
25
35
IB: Flag
IB: Myc
IP: Myc
IP: Flag
IP : Myc
IP: Flag
25
35
IB: Myc
IB: Flag
25
25
IB: HA
IB: HA
25
25
Input
35
IB: Myc
Input
180
IB: Myc
IB: Myc
IB: Flag
35
35
Input
Input
180
IB: Flag
35
IB: Flag
IB: Myc
IB: Flag
25
35
IB: Flag
IB: Myc

## Slide 6
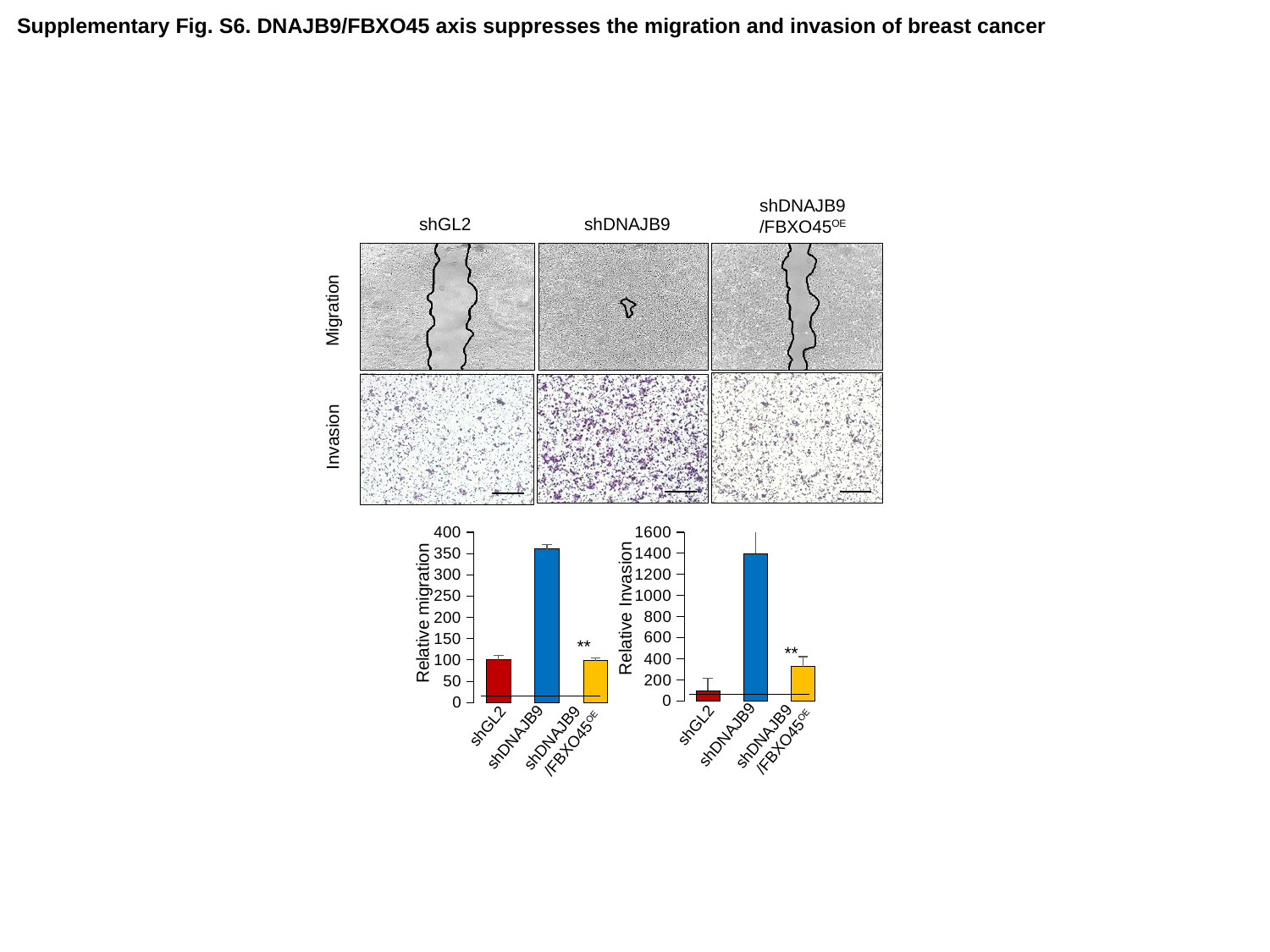

Supplementary Fig. S6. DNAJB9/FBXO45 axis suppresses the migration and invasion of breast cancer
shDNAJB9
/FBXO45OE
shDNAJB9
shGL2
Migration
Invasion
### Chart
| Category | |
|---|---|
### Chart
| Category | |
|---|---|**
shGL2
shDNAJB9
 /FBXO45OE
shDNAJB9
Relative Invasion
Relative migration
**
shGL2
shDNAJB9
 /FBXO45OE
shDNAJB9
